# Clinically relevant connectivity features define three subtypes of Parkinson's disease patients

**DOI:** 10.1002/hbm.25110

**Published:** 2020-06-26

**Authors:** Tao Guo, Xiaojun Guan, Cheng Zhou, Ting Gao, Jingjing Wu, Zhe Song, Min Xuan, Quanquan Gu, Peiyu Huang, Jiali Pu, Baorong Zhang, Feng Cui, Shunren Xia, Xiaojun Xu, Minming Zhang

**Affiliations:** ^1^ Department of Radiology, the Second Affiliated Hospital Zhejiang University School of Medicine Hangzhou China; ^2^ Department of Neurology, the Second Affiliated Hospital Zhejiang University School of Medicine Hangzhou China; ^3^ Department of Radiology Hangzhou Hospital of Traditional Chinese Medicine Hangzhou China; ^4^ Key Laboratory of Biomedical Engineering of Ministry of Education Zhejiang University Hangzhou China

**Keywords:** clustering analysis, heterogeneity, magnetic resonance imaging, Parkinson's disease, subtype

## Abstract

Parkinson's disease (PD) is characterized by complex clinical symptoms, including classic motor and nonmotor disturbances. Patients with PD vary in clinical manifestations and prognosis, which point to the existence of subtypes. This study aimed to find the fiber connectivity correlations with several crucial clinical symptoms and identify PD subtypes using unsupervised clustering analysis. One hundred and thirty‐four PD patients and 77 normal controls were enrolled. Canonical correlation analysis (CCA) was performed to define the clinically relevant connectivity features, which were then used in the hierarchical clustering analysis to identify the distinct subtypes of PD patients. Multimodal neuroimaging analyses were further used to explore the neurophysiological basis of these subtypes. The methodology was validated in an independent data set. CCA revealed two significant clinically relevant patterns (motor‐related pattern and depression‐related pattern; *r* = .94, *p* < .001 and *r* = .926, *p* = .001, respectively) among PD patients, and hierarchical clustering analysis identified three neurophysiological subtypes (“mild” subtype, “severe depression‐dominant” subtype and “severe motor‐dominant” subtype). Multimodal neuroimaging analyses suggested that the patients in the “severe depression‐dominant” subtype exhibited widespread disruptions both in function and structure, while the other two subtypes exhibited relatively mild abnormalities in brain function. In the independent validation, three similar subtypes were identified. In conclusion, we revealed heterogeneous subtypes of PD patients according to their distinct clinically relevant connectivity features. Importantly, depression symptoms have a considerable impact on brain damage in patients with PD.

## Graphical abstract

Parkinson's disease (PD) is a heterogeneous syndrome, which points to the subtypes in PD patients. This study used a data‐driven approach based on fiber connectivity to automatically identify subtypes in PD patients with distinct clinical features. Neuroimaging analyses revealed significant brain alterations behind each subtype.

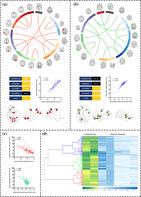

## 1 INTRODUCTION

Parkinson's disease (PD) has been recognized as a heterogeneous syndrome rather than just a dopaminergic motor disease (Titova, Padmakumar, Lewis, & Chaudhuri, 2017). The pathological and neurotransmitter basis of PD is not all dopaminergic, other transmitter systems such as cholinergic, noradrenergic, and serotonergic system are involved; nondopaminergic neurons, including the locus coeruleus and raphe area, are selectively vulnerable during the spreading of pathological alpha‐synuclein (Braak et al., 2003). These involvements lead to the heterogeneous clinical manifestations, not only including classic motor symptoms but involving differed extents of nonmotor symptoms (NMS). Over the past decades, a major emphasis has been placed on motor symptomatology; however, it is NMS burden that determines quality of life in PD patients (Barone et al., 2009). The whole NMS has a greater impact on health‐related quality of life than motor symptoms and NMS progression contributed importantly to quality of life decline in PD patients (Martinez‐Martin, Rodriguez‐Blazquez, Kurtis, Chaudhuri, & Group, 2011). Therefore, except for the motor symptoms, we need to pay attention to NMS when evaluating the disease severity of PD patients.

With such a highly heterogeneous syndrome, identifying the subtypes of PD is relevant as they may reflect different neurobiological basis and predict outcomes or responses to treatment. Many efforts to define subtypes have been made, including categorizing PD patients into age‐at‐onset categories, major motor subtypes, patterns of cognitive impairment, and specific nonmotor symptom‐dominant clinical subtypes (Marras & Chaudhuri, 2016; Sauerbier, Jenner, Todorova, & Chaudhuri, 2016; Schapira, Chaudhuri, & Jenner, 2017; Titova et al., 2017; Titova & Chaudhuri, 2018; Xuan et al., 2017). Other pioneering studies also divided PD patients into different subtypes using data‐driven analyses (Erro et al., 2016; Fereshtehnejad, Zeighami, Dagher, & Postuma, 2017; van Rooden et al., 2011). However, it is important to note that current subtyping approaches were mainly based on empirically clinical observation except some neurotransmitter dysfunction‐based nonmotor classifications that were supported by imaging and other biomarker studies (Titova et al., 2017; Titova & Chaudhuri, 2018). A natural attempt to describe subtypes using objective disease‐related features incorporating motor and nonmotor symptoms should be undertaken.

Magnetic resonance imaging (MRI) provides measures of various objective brain features. Fiber connectivity, derived from diffusion tensor imaging (DTI), is a relatively stable feature to reflect the brain attribution during a period of time, quantifying connectivity between a pair of brain regions in terms of fiber number (FN) and fractional anisotropy (FA). PD is associated with disrupted connectivity in the white matter network (Galantucci et al., 2017; Li et al., 2017; Wen, Heng et al., 2017; Wen, Xu et al., 2017), which suggests that fiber connectivity may serve as a disease‐related feature in multiple disease stages. Canonical correlation analysis (CCA), a data‐driven approach, enables investigation of the underlying relationships between two sets of variables (e.g., fiber connectivity and clinical symptoms) (Smith et al., 2015). Using CCA, we could identify the disease‐related brain features that simultaneously weighted several clinical symptoms. Unsupervised clustering analysis is a hypothesis‐free method that makes no use of labels to divide observations into homogenous subsets (Kimes, Liu, Neil Hayes, & Marron, 2017). Combined with objective disease‐related brain features, clustering analysis would help us better identify PD subtypes with stronger neurobiological correlations.

In the present study, we aimed to find the fiber connectivity correlations with several clinical symptoms using CCA and identify different PD subtypes using unsupervised clustering analysis that was based on objective fiber connectivity features. Multimodal neuroimaging analyses (including functional connectivity calculated from resting‐state functional MRI and microstructural alterations reflected by DTI) were used to deepen the neurophysiological basis behind these subtypes. We hypothesized that a specific fiber connectivity pattern was associated with definite clinical symptoms and that different subtypes defined by the clustering analysis would exhibit distinct neurophysiological foundations.

## 2 MATERIALS AND METHODS

### 2.1 Participants

All patients with PD and normal controls signed informed consent forms in accordance with the approval of the Medical Ethics Committee of the Second Affiliated Hospital of Zhejiang University School of Medicine.

A total of 134 PD patients (data set‐1) and 77 normal controls who underwent both structural T1 scanning and DTI scanning were recruited from August 2014 to November 2018. The diagnosis of PD was made by an experienced neurologist (B. Z.) according to UK Parkinson's Disease Society Brain Bank criteria (Hughes, Daniel, Kilford, & Lees, 1992). Normal controls and PD patients with a history of other neurologic or psychiatric disorders, brain trauma, or general exclusion criteria for MRI scanning were excluded from this study. For PD patients who were under antiparkinsonian treatment, clinical assessments and MRI scanning were performed in the morning after withdrawing all antiparkinsonian drugs overnight (at least 12 hr) (on “drug‐off status”) to minimize the potential pharmacological influences. Basic demographic information, such as age, sex and education, and neurologic and psychiatric scale assessments, including the unified Parkinson's disease rating scale (UPDRS), Hoehn‐Yahr stage, mini‐mental state examination (MMSE), Hamilton depression scale (HAMD), Hamilton anxiety scale (HAMA), Epworth Sleepiness Scale (ESS) and Parkinson's disease questionnaire (39 questions) (PDQ‐39), were obtained from all patients. For normal controls, basic demographic information, UPDRS motor score, HAMD score, HAMA score, and MMSE score were recorded. The mood status was evaluated for the past week to minimize the influences of fluctuation‐related acute mood symptoms. Given that patients with PD commonly and concurrently suffer from motor and nonmotor symptoms relating to overall disease progression and a decline in quality of life, an integrated score, that is, the global composite outcome (GCO), was calculated to evaluate overall disease severity (Fereshtehnejad et al., 2015; Fereshtehnejad et al., 2017). The GCO equally merged standardized z‐scores from different clinical domains, including motor (UPDRS III score), depression (HAMD score), anxiety (HAMA score), global cognition (MMSE score) and daytime sleepiness (ESS score) domains, which can account for different variations in the range or direction of different clinical scores and avoids overweighting a single clinical domain. A higher GCO score indicated worse function. The formula for the GCO was as follows:

1. $\mathrm{GCO}=\sum_{k=1}^{n}z_{k}$
2. $z_{k}=\left(\text{crud}e_{\text{score}}-\text{mean}\right)/\mathrm{standard}\;\mathrm{deviation}$

where *n* is the number of clinical domains and *z*_*k*_ is the standardized z‐score of a clinical domain.

### 2.2 MRI data acquisition

All participants were scanned on a 3.0‐Tesla MRI scanner (GE Discovery 750) equipped with an 8‐channel head coil. During MRI scanning, the head was stabilized using restraining foam pads, and earplugs were provided to reduce the noise. Structural T1 images were acquired using a fast spoiled gradient recalled sequence: repetition time = 7.336 ms; echo time = 3.036 ms; inversion time = 450 ms; flip angle = 11°; field of view = 260 × 260 mm^2^; matrix = 256 × 256; slice thickness = 1.2 mm; 196 continuous sagittal slices. DTI images were scanned using a spin echo‐echo planar imaging sequence with 30 gradient directions (b value = 1,000 s/m^2^): repetition time = 8,000 ms; echo time = 80 ms; flip angle = 90°; field of view = 256 × 256; matrix = 128 × 128; slice thickness = 2 mm; slice gap = 0 mm; 67 interleaved axial slices. Resting‐state fMRI (rs‐fMRI) images were acquired using a gradient recalled echo‐echo planar imaging sequence: repetition time = 2,000 ms; echo time = 30 ms; flip angle = 77°; field of view = 240 × 240 mm^2^; matrix = 64 × 64; slice thickness = 4 mm; slice gap = 0 mm; 38 interleaved axial slices.

### 2.3 DTI data analysis

#### 2.3.1 Preprocessing and fiber connectivity calculations

DTI images were preprocessed, and the structural network was constructed by the Pipeline for Analyzing braiN Diffusion imAges (PANDA) toolbox (http://www.nitrc.org/projects/panda/) (Cui, Zhong, Xu, He, & Gong, 2013), which incorporates the FMRIB Diffusion toolbox (http://www.fmrib.ox.ac.uk/fsl/) and Diffusion Toolkit software (http://trackvis.org/dtk/). The preprocessing procedures included the following steps: (1) brain extraction; (2) correction for eddy‐current‐induced distortion and simple head‐motion artifacts; and (3) diffusion parameter calculation (i.e., fractional anisotropy [FA], mean diffusivity [MD]).

**FIGURE 1:**
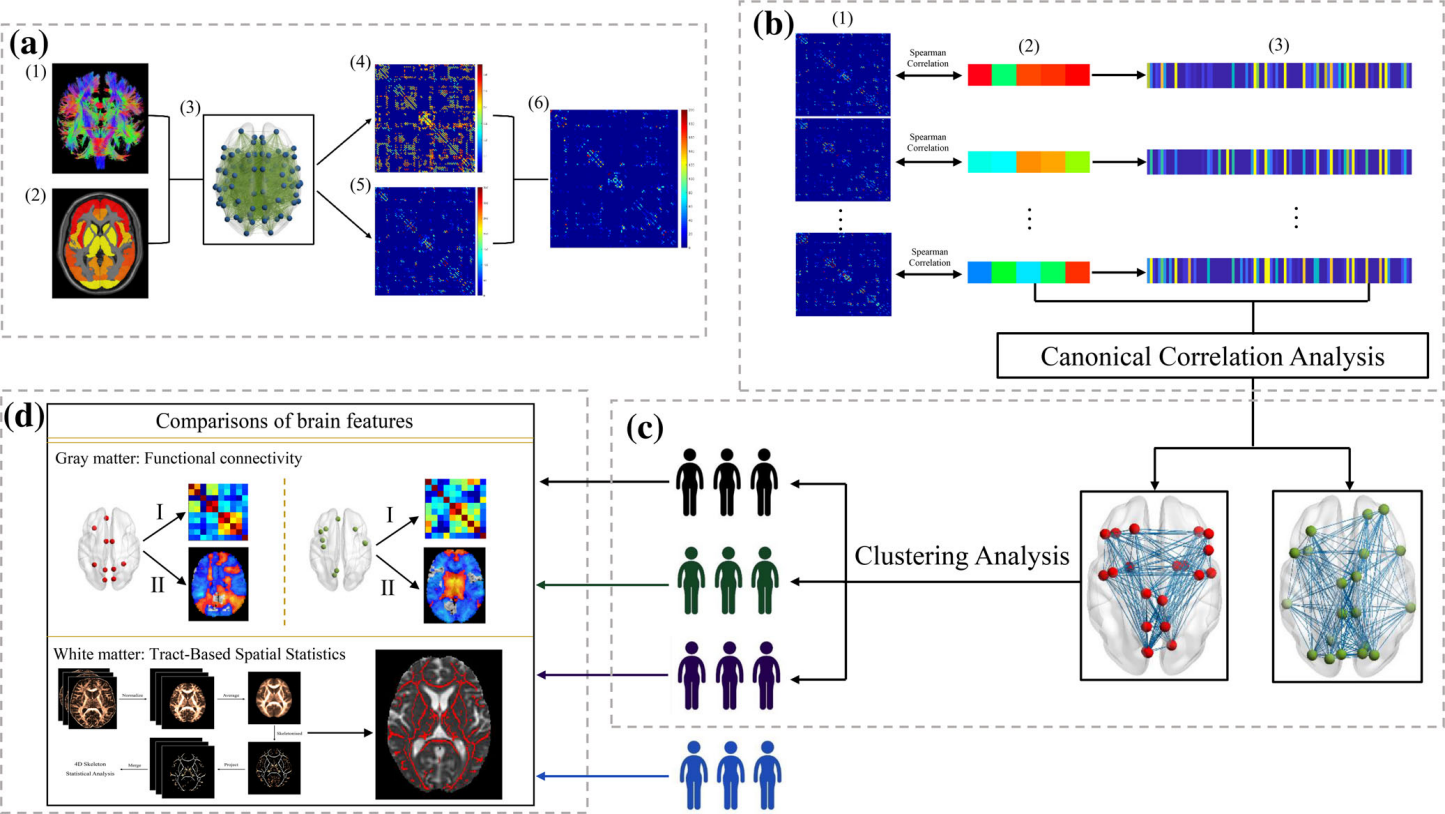
Data analysis schematic and workflow. (a) Individual network construction. (1) Deterministic tractography based on FA images in native space and (2) HOA parcellation in native space were used to construct the (3) structural network. (4) The FA‐weighted matrix and (5) FN‐weighted matrix were simultaneously generated. (6) The final matrix representing fiber connectivity was calculated by multiplying FN and FA along the fiber bundles connecting a pair of nodes. (b) Identification of clinically relevant connectivity features. (1) Brain‐wide fiber connectivity was correlated with (2) clinical scores to get (3) significant connectivity features; CCA was conducted based on (2) clinical scores and (3) significant connectivity features to identify a low‐dimensional representation of those connectivity features. (c) Clustering analysis in whole PD group. Clustering analysis based on two clinically relevant connectivity patterns identified three subtypes in PD (colored by dark, green, and purple cartoons). (d) Neuroimaging analyses for PD subtypes and normal controls (colored by blue cartoons). CCA, canonical correlation analysis; FA, fractional anisotropy; FN, fiber number; HOA, Harvard‐Oxford cortical and subcortical atlas

The DTI‐based structural network was constructed as in a previous study (Figure 1a) (Guan et al., 2019), where nodes represented brain regions and edges represented interregional white matter tracks. The Harvard‐Oxford cortical and subcortical atlas (HOA) with 110 regions of interest (ROIs) was used to define network nodes. Deterministic tractography was performed to obtain interregional white matter tracts. Specifically, the fiber assignment continuous tracking (FACT) algorithm was applied to reconstruct interregional tracts. Tractography was terminated if it turned at an angle >45° or reached a voxel with an FA < 0.2. This procedure simultaneously generated the FN and FA connectivity matrixes.

To reduce the influence of pseudoconnections, a threshold of fiber numbers, *n* = 3, was set to ensure the major connections among cortical regions (Shu et al., 2009). Considering that fiber connectivity between a pair of nodes is affected by the number of fiber bundles as well as the fiber integrity, independent measures of FN or FA could not reflect the actual connectivity. Therefore, we multiplied FN by the mean FA along the fiber bundles connecting pairs of nodes to represent the fiber connectivity. This strategy more comprehensively revealed the white matter structure (Lo et al., 2010).

#### 2.3.2 CCA and clustering analysis

Each participant's 110 × 110 fiber connectivity matrix contained 5,995 (110 × 109/2) unique connectivity features, necessitating a procedure for selecting a subset of relevant and nonredundant connectivity features. To select these clinically relevant connectivity features, we first used Spearman's rank correlations to identify connectivity features that were significantly correlated (*p* < .005) with clinical symptom severity, including motor symptoms (UPDRS III score) and several NMS (global cognition, MMSE score; depression, HAMD score; anxiety, HAMA score; daytime sleepiness, ESS score). Then, we carried out a multivariate analysis named CCA to define a low‐dimensional representation of those connectivity features (Figure 1b). Each significant CCA mode identified a linear combination of a set of original variables (*X*, for example, clinical symptoms) and a linear combination of another set of original variables (*Y*, for example, connectivity features), where the correlation between these two combinations was maximal. These two combinations represented the canonical variables for each set and were termed the clinical component *(U)* and connectivity pattern *(V)*, respectively. Canonical loading describes the association between an original variable and its corresponding canonical variable. Cross loading depicts the correlation between an original variable and the canonical variable of another set. Squared loading indicates the amount of variance of a variable explained by the canonical variate (Sherry & Henson, 2005; Smith et al., 2015) (the detailed procedure is shown in Figure S1). This data‐driven approach enabled us to find two modes that related sets of fiber connectivity features to sets of clinical symptom measures.

Clustering analysis was performed in R with the cluster package. We used hierarchical clustering to assign subjects to nested subgroups with similar patterns of abnormal connectivity along these two CCA modes of connectivity features (Figure 1c). The Euclidean distance between every pair of participants in this two‐dimensional connectivity feature space was calculated, and then, Ward's minimum variance method was used to iteratively link pairs of participants in closest proximity, forming progressively larger clusters in a hierarchical tree. Next, we used the NbClust package to determine the best number of clusters in our analysis.

#### 2.3.3 Tract‐based spatial statistics analysis

To explore the white matter microstructural alterations underlying different subtypes, FA and MD maps were used to reflect the microstructure of brain‐wide white matter in PD subtypes. The tract‐based spatial statistics (TBSS) method was used to extract the main fibers across the whole brain according to the following steps: (1) individual FA images were aligned to the standard space template using nonlinear registration; (2) the mean FA image was calculated and compressed to form a mean skeleton representing topological features of all tracts derived from the whole group, and an FA threshold of 0.2 was set to remove trivial tracts; and (3) each subject's aligned FA images were projected onto the fiber skeleton template for statistical analysis. MD maps were also normalized to the skeleton using TBSS.

### 2.4 Rs‐fMRI data analysis: Preprocessing and functional connectivity analysis

The rs‐fMRI data preprocessing was performed using the Statistical Parametric Mapping version 12 (SPM, https://www.fil.ion.ucl.ac.uk/spm/) and Data Processing & Analysis for (Resting‐State) Brain Imaging suite (http://rfmri.org/dpabi) according to a standard pipeline (Yan, Wang, Zuo, & Zang, 2016). Of note, 13 patients and 1 normal control were excluded because of the poor image quality. Finally, 121 PD patients and 76 normal controls were included in the functional connectivity analysis.

CCA revealed two distinct connectivity patterns, each pattern comprised a set of nodes. To uncover the neurophysiological substrates of the subtypes defined by the unsupervised clustering analysis, we firstly calculated a functional connectivity matrix to reflect the local function of each pattern (Figure 1d‐I). Then, to explore the global function of each pattern, we calculated the functional connectivity outside of each pattern (Figure 1d‐II). Fisher's r‐to‐z transformation was applied to improve data distributions for parametric statistical analyses. A detailed description of rs‐fMRI data analysis was shown in the Supplementary Materials.

### 2.5 Statistical analysis

Statistical analyses of demographic and clinical data were performed using SPSS 19.0 statistical software. The one‐sample Kolmogorov–Smirnov test was used to check the data normality. Differences in the age, education, sex distribution, and clinical symptom scores between groups were compared with the unpaired *t*‐tests, the Mann–Whitney U tests, and the Pearson chi‐squared test as appropriate. Paired *t*‐tests were used to compare the normalized UPDRS III scores and HAMD scores to determine the predominant clinical symptom in a specific subtype. Statistical significance was set at *p* < .05.

Statistical analyses of functional connectivity were conducted using unpaired *t* tests with age, sex, and education as covariates. Multiple comparison corrections were performed using the false discovery rate (FDR) correction with q < .05. The intergroup comparisons of the white matter microstructure (FA and MD skeletons) were performed using the *Randomized* script in the FMRIB Software Library (https://fsl.fmrib.ox.ac.uk/fsl/fslwiki/), with age, sex, and education as covariates. Permutation tests with 5,000 iterations and threshold‐free cluster enhancement with a threshold of corrected *p* < .05, corrected for multiple comparisons, were performed in the intergroup comparisons.

### 2.6 Independent validation of the subtypes

To validate the subtypes we defined in data set‐1, we performed the same data‐driven procedures in an independent data set (data set‐2) that included 98 PD patients who were recruited from December 2018 to October 2019. Specifically, CCA and hierarchical clustering analysis were conducted again to identify the PD subtypes.

## 3 RESULTS

### 3.1 Clinically relevant fiber connectivity patterns in Parkinson's disease

**FIGURE 2:**
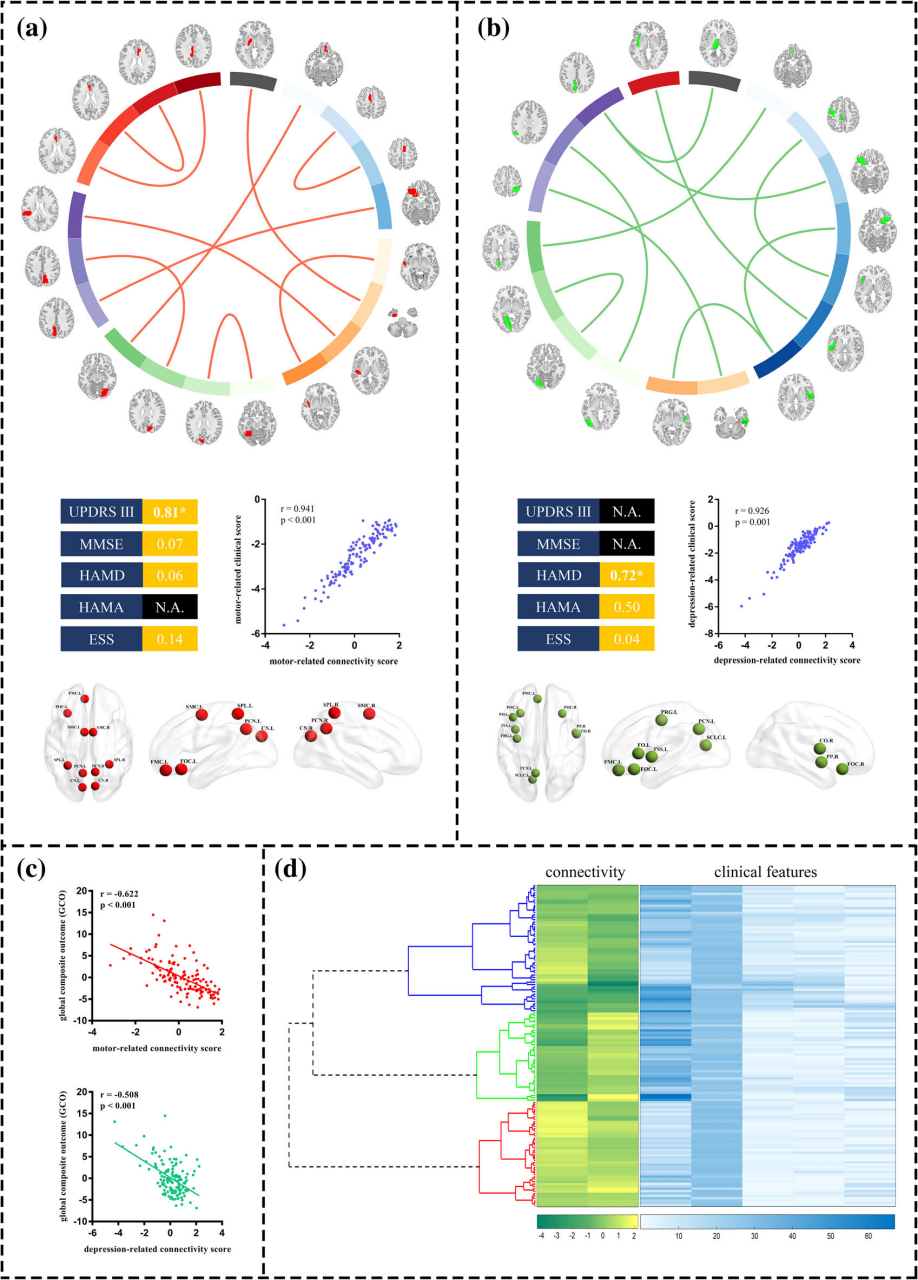
CCA and hierarchical clustering define three connectivity‐based subtypes for PD. CCA was used to define a low‐dimensional representation of clinically relevant connectivity features and identified a “motor‐related” pattern (a) and a “depression‐related” pattern (b), represented by linear combinations of connectivity features (connectivity component) that were correlated with linear combinations of symptoms (clinical component). The circles in (a) and (b) depict the connectivity features (top 10) that were most highly correlated with each clinical component. The scatterplots in (a) and (b) illustrate the correlation between the connectivity component and clinical component for the motor‐related pattern (*r* = .941, *p* < .001) and depression‐related pattern (*r* = .926, *p* = .001), respectively. To the left of each scatterplot, squared cross loadings for clinical scores are depicted. N.A. = not significant. For visualization, below each scatterplot, squared canonical loadings for connectivity features are summarized by depicting the neuroanatomical distribution of the top 10 ROIs with the largest R^2^ values, summed across all connectivity features associated with a given node (neuroanatomical distribution of the top 20% ROIs with the largest *R* ^2^ values is presented in Figure S2). (c) Correlations between clinically relevant connectivity scores and overall disease severity (GCO). (d) Hierarchical clustering analysis. According to the connectivity scores, patients with PD were assigned into three subtypes, whose connectivity scores and clinical features are presented as green diagrams and blue diagrams, respectively. CCA, canonical correlation analysis; GCO, global composite outcome; ROI, region of interest

CCA revealed two significant modes that related sets of fiber connectivity features (connectivity pattern) to sets of clinical symptom measures (clinical component) (Figure 2a,b). For each connectivity pattern, squared canonical loadings for each connectivity feature were summarized by depicting the neuroanatomical distribution of the top 10 ROIs with the largest *R* ^2^ values, summed across all connectivity features associated with a given node. The first mode defined a connectivity pattern predominantly related to brain nodes close to the midline, including the bilateral precuneus cortex, cuneal cortex, supplementary motor cortex (SMC), and superior parietal lobule (SPL), that particularly account motor symptoms (squared canonical loadings = 0.81; Figure 2a). We termed this CCA mode a motor‐related pattern. The second mode defined a set of connectivity features predominantly related to the lateral limbic nodes, including the insula, frontal orbital cortex, frontal and central operculum cortex, that mostly explained depression (squared canonical loadings = 0.72; Figure 2b). We called this CCA mode a depression‐related pattern. Moreover, both the motor‐related connectivity pattern and depression‐related connectivity pattern were associated with overall disease severity (GCO; Figure 2c). In summary, CCA identified two disease‐related connectivity patterns that were especially related to motor function and depression.

### 3.2 Clinically relevant fiber connectivity patterns define three subtypes of Parkinson's disease

We tested whether these fiber connectivity patterns tended to cluster patients into subgroups. We used hierarchical clustering to discover clusters of patients by assigning them to nested subgroups with similar fiber connectivity patterns. This unsupervised approach objectively determined 15 clusters and 3 clusters as the optimal solutions. Here, we prioritized three clusters defined by distinct fiber connectivity features, which resulted in a better‐balanced data distribution and ensured the statistical power to detect biologically meaningful differences (Figure 2d).

### 3.3 Clinical profiles of subtypes defined by the connectivity patterns

Demographic information and clinical scores are shown in Table 1. No significant differences in age, sex, or education were found among the three PD clusters. In comparison to the normal controls, Cluster 2 had a different sex distribution, and both Cluster 1 and Cluster 2 exhibited lower education.

**TABLE 1:** Demographic information and clinical scale scores of all participants

	Parkinson's disease patients			Normal controls (NCs, *n* = 77)	Comparisons						
	Cluster 1 (S‐depression, *n* = 53)	Cluster 2 (S‐motor, *n* = 37)	Cluster 3 (mild, *n* = 44)		Comparisons among PD groups (*p* value)	Post hoc (*p* value)			Parkinson's disease subtypes versus NCs (*p* value)		
						Cluster 1 versus Cluster 2	Cluster 1 versus Cluster 3	Cluster 2 versus Cluster 3	Cluster 1 versus NCs	Cluster 2 versus NCs	Cluster 3 versus NCs
**Sex (M/F)**	29/24	26/11	24/20	33/44	.259	–	–	–	.183	.006	.215
**Age**											
Mean ± *SD* (min, max)	60.89 ± 8.68 (41.17, 82.31)	63.34 ± 10.05 (42.49, 85.11)	59.38 ± 8.30 (39.69, 75.66)	60.22 ± 7.40 (47.72, 83.15)	.142	–	–	–	.635	.064	.569
**Education**											
Mean ± *SD* (min, max)	7.49 ± 4.08 (0, 16)	7.38 ± 5.21 (0, 18)	9.15 ± 4.47 (1, 18)	10.26 ± 3.70 (1, 16)	.129	–	–	–	<.001	.002	.142
**Duration**											
Mean ± *SD* (min, max)	3.86 ± 4.14 (0.08, 26.37)	4.86 ± 3.03 (0.57, 12.03)	3.77 ± 3.58 (0.29, 20.22)	–	.343	–	–	–	–	–	–
**LED**											
Mean ± *SD* (min, max, median)	307.92 ± 282.41 (0, 999, 287.5)	397.70 ± 358.55 (0, 1,300, 375)	306.25 ± 245.12 (0, 825, 343.75)	–	.285	–	–	–	–	–	–
**GCO**											
Mean ± *SD* (min, max, median)	1.94 ± 4.13 (−3.86, 14.48, 1.19)	0.66 ± 2.46 (−5.63, 5.35, 0.52)	−2.89 ± 1.93 (−6.89, 2.82, −2.86)	–	<.001	–	<.001	<.001	–	–	–
**PDQ‐39**											
Mean ± *SD* (min, max, median)	30.04 ± 18.72 (4, 85, 28)	32.46 ± 21.45 (4, 73, 35)	17.89 ± 17.61 (0, 82, 13.5)	–	<.001	.581	.002	.002	–	–	–
**UPDRS III**											
Mean ± *SD* (min, max, median)	27.66 ± 12.08 (8, 55, 27)	35.89 ± 11.71 (17, 67, 35)	14.95 ± 6.80 (5, 34, 13.5)	0.62 ± 1.18 (0, 5, 0)	<.001	.002	<.001	<.001	–	–	–
**HY**											
Median, range	2, 1–3	2, 1.5–4	1, 1–3	–	<.001	.006	<.001	<.001	–	–	–
**MMSE**											
Mean ± *SD* (min, max, median)	26.72 ± 3.63 (15, 30, 27)	25.70 ± 3.80 (15, 30, 27)	27.73 ± 3.53 (13, 30, 28.5)	28.25 ± 1.99 (21, 30)	.001	.126	.008	.001	.001	<.001	.841
**HAMD**											
Mean ± *SD* (min, max, median)	10.21 ± 6.07 (2, 31, 9)	4.16 ± 2.83 (0, 13, 4)	2.98 ± 1.87 (0, 7, 3)	2.30 ± 2.91 (0, 17, 1)	<.001	<.001	<.001	.034	<.001	<.001	.008
**HAMA**											
Mean ± *SD* (min, max, median)	8.89 ± 5.62 (1, 25, 8)	3.76 ± 3.01 (0, 12, 3)	2.98 ± 2.26 (0, 10, 3)	3.48 ± 4.16 (0, 22, 2)	<.001	<.001	<.001	.188	<.001	.178	.446
**ESS**											
Mean ± *SD* (min, max, median)	5.43 ± 5.15 (0, 24, 4)	7.78 ± 5.57 (0, 19, 7)	4.09 ± 4.30 (0, 15, 2)	–	.006	.042	.149	<.001	–	–	–

Abbreviations: ESS, epworth sleepiness scale; GCO, global composite outcome; HAMA, Hamilton anxiety scale; HAMD, Hamilton depression scale; HY, Hoehn‐Yahr stage; LED, levodopa equivalent dose; MMSE, mini‐mental state examination; NCs, normal controls; PDQ‐39, Parkinson's disease questionnaire (39 questions); UPDRS III, unified Parkinson's disease rating scale‐motor.

Of the three clusters, we found one cluster that was characterized by slightly lower overall disease impairment (Cluster 3: lower GCO); one cluster that was characterized by severe overall disease impairment with depressive symptoms as dominant (Cluster 1: higher GCO, higher normalized depression score, *p* = .001; Table S1); and the remaining cluster that was characterized by severe overall disease impairment with predominant motor disturbances (Cluster 2: higher GCO, higher normalized motor score, *p* < .001; Table S1). Therefore, we termed Cluster 1 as the “severe depression‐dominant” subtype (S‐depression), Cluster 2 as the “severe motor‐dominant” subtype (S‐motor), and Cluster 3 as the “mild” subtype.

### 3.4 Functional connectivity within the clinically relevant fiber connectivity pattern

**FIGURE 3:**
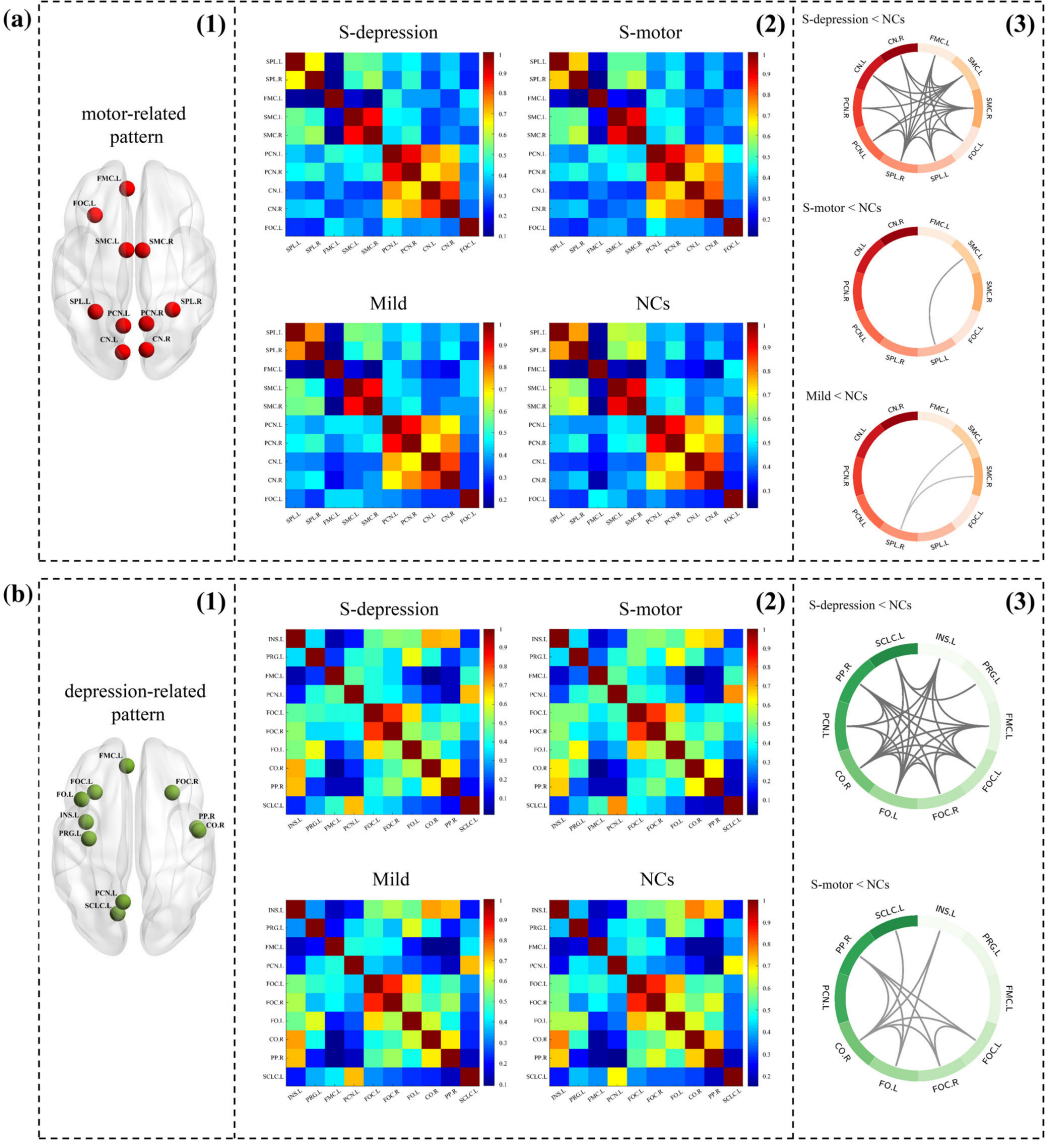
Subtype differences in functional connectivity within the motor‐related pattern (a) and the depression‐related pattern (b). The group mean functional connectivity matrix is presented in column (2). Comparison results are shown in column (3) (corrected by false discovery rate (FDR) with q < 0.05): the gray curve between each pair of ROIs represented the reduced functional connectivity in patients with PD compared with the normal controls. The S‐depression subtype showed widespread disrupted functional connectivity both within the motor‐related pattern and depression‐related pattern. The S‐motor subtype exhibited slightly decreased functional connectivity in the motor‐related pattern and moderately decreased functional connectivity in the depression‐related pattern. The mild subtype only showed slightly lower functional connectivity in the motor‐related pattern. SPL.L, left superior parietal lobule; SPL.R, right superior parietal lobule; FMC.L, left frontal medial cortex; SMC.L, left supplementary motor cortex; SMC.R, right supplementary motor cortex; PCN.L, left precuneus; PCN.R, right precuneus; CN.L, left cuneus; CN.R, right cuneus; FOC.L, left frontal orbital cortex; FOC.R, right frontal orbital cortex; INS.L, left insula; PRG.L, left precentral gyrus; FO.L, left frontal operculum; CO.R, right central operculum; PP.R, right planum polare; SCLC.L, left supracalcarine cortex

To reveal the local function of the connectivity pattern derived from CCA, we calculated functional connectivity among the top 10 ROIs within each connectivity pattern in each group. The mean functional connectivity matrix for each group is shown in Figure 3 (column 2). Comparisons of local function between groups are presented in Figure 3 (column 3, corrected by false discovery rate (FDR) with q < 0.05): the gray curve between each pair of ROIs represents the reduced functional connectivity in PD patients compared to the normal controls. Compared with the normal controls, in the motor‐related pattern, the mild and S‐motor subtypes showed slightly decreased functional connectivity, while the S‐depression subtype exhibited widespread disrupted functional connectivity (Figure 3a, column(3)), indicating that the S‐depression subtype was associated with poor function within the motor‐related pattern. In the depression‐related pattern, the S‐depression subtype showed widespread disruption in functional connectivity among ROIs, and the S‐motor subtype showed moderately decreased functional connectivity among ROIs in comparison to normal controls (Figure 3b, column(3)). There was no difference between mild subtype and normal controls in terms of functional connectivity among the ROIs in the depression‐related pattern. Details about the decreased functional connectivity among the ROIs are presented in Table S2.

### 3.5 Functional connectivity outside of the clinically relevant fiber connectivity pattern

**FIGURE 4:**
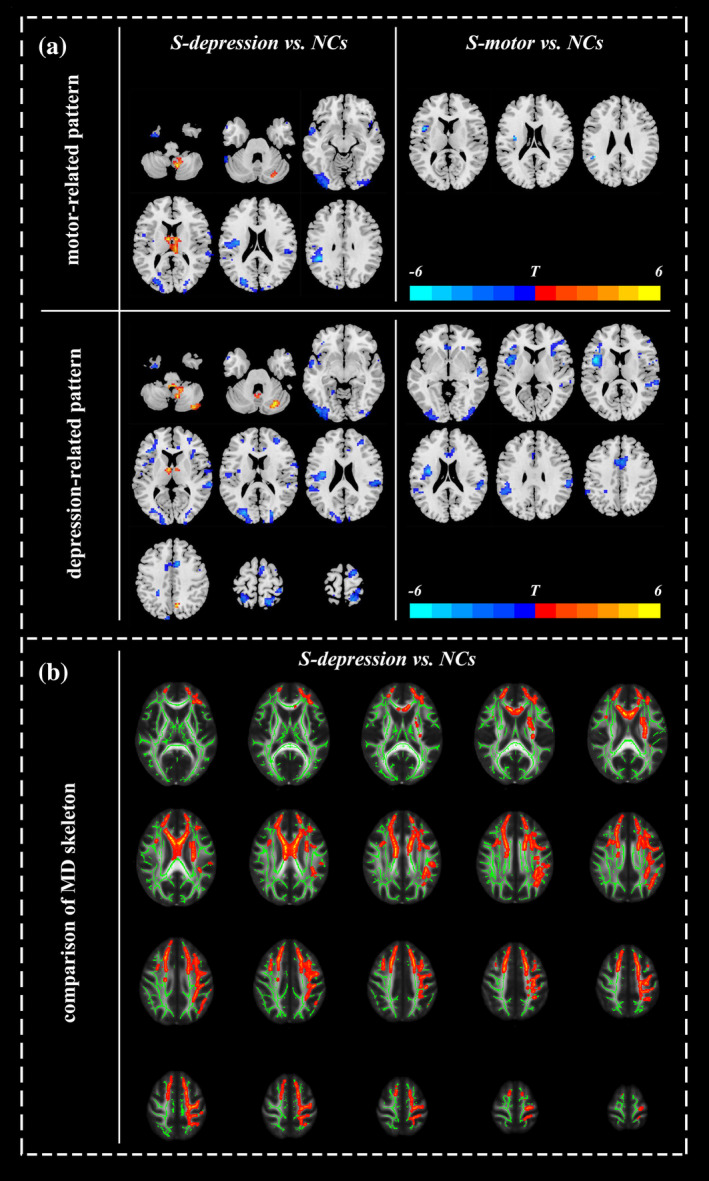
Functional connectivity alterations between the clinically relevant connectivity pattern and the remaining portions of the brain (a) and microstructural changes (b) in the three subtypes of PD. (a) For both the motor‐related and depression‐related patterns, the S‐depression subtype showed widespread decreased functional connectivity across the whole brain, mainly involving the frontal‐temporal and parietal‐occipital regions and increased functional connectivity in the cerebellum and thalamus. The S‐motor subtype showed decreased functional connectivity between the motor‐related pattern and the inferior frontal as well as the supramarginal gyri, dysconnectivity between the depression‐related pattern and widespread brain regions covering the frontal‐temporal, parietal‐occipital and limbic regions. The results were corrected by false discovery rate (FDR) with q < 0.05 and cluster size >10 voxels. (b) Compared with NCs, the S‐depression subtype showed increased MD in widespread brain regions mainly involving the superior longitudinal fasciculus, corona radiata, corpus callosum, forceps minor, and uncinate fasciculus (*p* < .05, corrected by TFCE). No difference was observed among the other groups

To detect the global function of the connectivity pattern, we used a seed‐based approach to explore the functional connectivity between the connectivity pattern and the remaining voxels in the brain. For the motor‐related pattern, the S‐depression subtype showed widespread decreased functional connectivity across the whole brain, mainly involving the frontal‐temporal, parietal‐occipital, and cingulate regions, while the S‐motor subtype showed dysconnectivity in the inferior frontal and supramarginal gyri compared with the normal controls. Moreover, the S‐depression subtype exhibited increased functional connectivity between the motor‐related pattern and the cerebellum as well as the thalamus compared with the normal controls (FDR‐corrected, q < 0.05, cluster size >10 voxels; Figure 4a, Table S3).

Regarding the depression‐related pattern, in comparison to the normal controls, both the S‐depression and S‐motor subtypes exhibited widespread decreased functional connectivity in the whole brain covering the frontal‐temporal, parietal‐occipital and limbic regions, while the S‐depression subtype also showed increased functional connectivity in the cerebellum, thalamus, and precuneus (FDR‐corrected, q < 0.05, cluster size >10 voxels; Figure 4a, Table S4). No difference in functional connectivity with either the motor‐related pattern or depression‐related pattern was observed between the mild subtype and normal controls.

### 3.6 Microstructural changes in the three subtypes of Parkinson's disease

Compared with normal controls, only the S‐depression subtype showed increased MD in widespread brain regions, which mainly included the superior longitudinal fasciculus, corona radiata, corpus callosum, forceps minor, and uncinate fasciculus (*p* < .05, corrected by TFCE; Figure 4b, Table S5). No difference in the MD skeleton was observed among the other pairs of comparisons, and no significant differences in the FA skeleton were observed among the groups.

### 3.7 Robustness of subtypes in Parkinson's disease

We used the same data‐driven procedures in another independent data set (data set‐2) to validate the robustness of the subtypes we defined in data set‐1. First, CCA stably revealed a depression‐related pattern and motor‐related pattern (Figure S3, A and B), and these two clinically relevant connectivity patterns mostly explained depression and motor symptoms, respectively (squared cross loading = 0.468 and 0.279, respectively). In addition to the depression‐ and motor‐related patterns, another component that mainly explained daytime sleepiness was found (Figure S3, C). This inconsistency across the two CCA procedures might have resulted from the heterogeneous clinical manifestations of the patients between the two data sets (Table S6; PD patients in data set‐2 had mild motor impairments). Then, taking the same approaches as before, the motor‐ and depression‐related connectivity patterns were used in the hierarchical clustering. This procedure also determined three clusters as the optimal solution (Figure S3, D).

Demographic information and clinical scores for three clusters in data set‐2 are shown in Table S7. No significant differences in age, sex, or education were found among the three clusters. Similarly, of the three clusters, we found one cluster that was characterized by slightly lower overall disease impairment (Cluster 1: lower GCO); one cluster that was characterized by severe overall disease impairment with depressive symptoms as dominant (Cluster 3: normalized motor score < normalized depression score, *p* < .001); and the remaining cluster was characterized by severe overall disease impairment with a trend of a predominant motor disturbance (Cluster 2: normalized motor score > normalized depression score, *p* = .066). In summary, by employing this independent data set, we found three different subtypes (mild, S‐motor, and S‐depression) that were similar to what we previously found, therefore, verifying the robustness of these subtypes.

## 4 DISCUSSION

In the present study, we found significant fiber connectivity patterns with significant clinical relevance and explored heterogeneous PD subtypes using unbiased objective data‐driven approaches and further revealed the potential structural and functional underpinnings behind these subtypes. The main findings were as follows: (a) motor‐related and depression‐related fiber connectivity patterns were observed in PD patients, both of which correlated with overall disease severity (GCO score); (b) unsupervised clustering analysis using objective connectivity features defined three PD subtypes with very different clinical profiles; (c) the S‐depression subtype showed widespread disruptions both in function and structure, while the other two subtypes exhibited relatively mild brain abnormalities in functional connectivity; and (d) these three subtypes were robustly identified, as validated by an independent data set.

### 4.1 Distinct clinically relevant fiber connectivity patterns in Parkinson's disease

PD is generally known as a movement disorder, there is an increasing awareness of the nonmotor manifestations in PD as they often present before PD diagnosis and contribute to impaired quality of life, which indicates that evaluating PD severity only depending on motor symptoms is one‐sided. In this study, we considered motor symptoms and several nonmotor symptoms together and extracted two clinically relevant connectivity patterns that were most associated with motor symptoms and depressive symptoms. Both of these clinically relevant patterns were associated with overall disease severity. In clinical practice, motor impairments have been recognized as a prominent component of PD and are the core criteria for PD diagnosis (Hughes et al., 1992; Postuma et al., 2015), which emphasizes the centrality of motor disturbances in the clinical profiles of PD patients. In our study, we identified a motor‐related pattern based on various clinical manifestations and further confirmed the centrality of motor symptoms in PD, which was in line with previous evidence. Moreover, it is now widely realized that PD evolves into a multisystem disorder that is accompanied by a wide variety of nonmotor symptoms, some of which may be present before the onset of motor features (Schapira et al., 2017; Titova et al., 2017). Depression, a common nonmotor symptom, could appear in the prodromal phase of PD and has been shown to nearly double an individual's risk of subsequently developing PD (Noyce et al., 2012). Furthermore, depression had a strong association with health‐related quality of life in PD patients (Gallagher, Lees, & Schrag, 2010), which implied the importance of depressive symptoms in PD. In our study, a depression‐related pattern was automatically extracted among several nonmotor symptoms, which further demonstrated that depression was a considerable manifestation in PD. In brief, our study suggested that the core features of PD include motor impairment and depression.

In the motor‐related connectivity pattern, the regions were predominantly located in the medial brain involving the bilateral precuneus cortex, cuneal cortex, SPL, and SMC, as well as the left medial frontal cortex and orbitofrontal cortex. Although most associated with motor symptoms, this connectivity pattern was also related to other nonmotor symptoms, including global cognition, depression, and excessive daytime sleepiness. Similarly, previous researches have revealed that disrupted structure or function in medial brain regions, including the frontal, parietal and occipital cortices (e.g., SMC, precuneus, and SPL), not only impacted the parkinsonian motor system (C. Huang et al., 2013; Jenkins, Jahanshahi, Jueptner, Passingham, & Brooks, 2000; Timmermann et al., 2003; Wilson, Niccolini, Pellicano, & Politis, 2019; Wu et al., 2009) but also related to some nonmotor symptoms, such as cognitive impairment, depression and sleep disturbance (Chondrogiorgi et al., 2016; C. Huang et al., 2007; Kato et al., 2012; Morgan, Ledbetter, Ferrier, Zweig, & Disbrow, 2018; Wen et al., 2016). This evidence indicated that nodes in medial brain regions participated in the generation of various clinical symptoms, including both motor and nonmotor symptoms, and may act as key nodes in the whole brain. The human cerebral cortex is organized into a complex network of local circuits and long‐range fiber pathways. This complex network forms the structural substrate for distributed interactions among specialized brain regions (Passingham, Stephan, & Kotter, 2002; Sporns, Tononi, & Kotter, 2005). Notably, Hagmann and colleagues revealed a “structural core” within the posterior medial and parietal cortical regions as well as the frontal cortex (e.g., precuneus, cuneus, superior parietal cortex, and medial frontal cortex) (Hagmann et al., 2008). Brain regions in this “structural core” shared high centrality and showed a close relationship with function, which suggested a central role of the regions within this “structural core” in functional integration that was required for the generation of behavior. Interestingly, the distribution of brain regions in our motor‐related pattern was similar to the pattern of this “structural core.” The significant associations between motor‐related connectivity pattern and the various clinical symptoms further demonstrated a correspondence between this core structural connectivity feature and the clinical behavior manifestations. Combined with our results, we suppose that this motor‐related connectivity pattern is a core feature of PD that reflects the overall clinical manifestations with motor impairment as the cardinal symptom.

Depression is a syndrome of stress and emotion dysregulation and can affect up to 10–45% of PD patients. It often predates motor symptoms by several years and belongs to nonmotor features that may herald the development of PD (Burn, 2002; Ishihara & Brayne, 2006). In the present study, we identified a depression‐related pattern mainly located in the frontal‐limbic network that included the insula, orbitofrontal cortex, frontal and central operculum cortex. The insula has a well‐established role in processing affection and emotion, and the orbitofrontal cortex promotes integrating and evaluating multimodal information and making decisions (P. Huang et al., 2016). Damage to these regions disrupts social behaviors and emotional processing (Bechara, Damasio, & Damasio, 2000). A significant biological basis for depression in PD is the outcome of any damage to limbic noradrenergic and dopaminergic mechanisms (Remy, Doder, Lees, Turjanski, & Brooks, 2005). Previous studies have also revealed that depression in PD was associated with dysfunction in frontal‐limbic brain networks (Coulter, Ibrahimi, Patel, & Agius, 2017; Drysdale et al., 2017; Gujral, Aizenstein, Reynolds 3rd, Butters, & Erickson, 2017), which was in line with our findings. Considering the strong association between connectivity scores and depressive symptoms, we speculate that this depression‐related pattern, mainly composed of limbic regions, may act as an objective marker for depression in PD patients.

### 4.2 Fiber connectivity defined three distinct subtypes of Parkinson's disease

The fiber connectivity‐based clustering analysis revealed three PD subtypes with distinct clinical profiles, named mild, S‐motor, and S‐depression, respectively. The mild subtype exhibited mild impairment in overall disease severity as well as in all clinical domains and was widely observed in several studies (Erro et al., 2013; Mu et al., 2017; van Rooden et al., 2011). Given that PD patients are very heterogeneous in terms of progression, patients in this subtype may show a relatively slow progression (Erro et al., 2013). The S‐motor and S‐depression subtype showed severe disease status, but with a divergence in symptomatic expression: S‐motor corresponding to the traditional motor‐dominant view of PD, showing severe motor impairment, which was consistent with previous findings (Erro et al., 2013; Mu et al., 2017); S‐depression representing a specific nonmotor dominant subtype also described in clinical studies (Sauerbier et al., 2016). Notably, depression in PD may manifest in two clinical phenotypes: anxious‐depressed and depressed, which could show different clinical features (Brown et al., 2011; Burn et al., 2012). In our study, we identified a depression‐dominant phenotype that comorbidity of higher anxious scores, corresponding to the “anxious‐depressed” phenotype proposed by Brown and colleagues. This specific phenotype may be characterized by a distinct pathophysiological mechanism that needs to be considered in future treatment. As a result, the identified subtypes in the present study showed a closely corresponding with previous studies, which provided the evidence for the existence of these subtypes. Importantly, the subtypes in this study were defined by objective brain features and thus, reducing the influence of clinical scale assessment on subtyping results.

### 4.3 Depression has a considerable impact on brain function and structure in Parkinson's disease revealed by neuroimaging analyses

To reveal the neural basis underlying these distinct subtypes, we used multimodal MRI to detect alterations in local and global function of the clinically relevant connectivity patterns as well as white matter structural alterations among these three subtypes.

Regarding the local function of the motor‐related pattern, we observed that the mild subtype with relatively prominent motor impairments exhibited decreased functional connectivity between the bilateral SMC and right SPL. The SMC has been suggested to be critical in planning and initiating movements (Jenkins et al., 2000). In PD patients, hypoactivation in the SMC during tasks requiring motor selection and initiation has been extensively reported (Buhmann et al., 2003; Haslinger et al., 2001; Rascol et al., 1997). In addition, previous works have discovered disrupted neural activity and metabolism in the SMC and parietal cortex in PD patients (Eckert et al., 2005; Wu et al., 2009). Therefore, among the functional connectivity between each pair of nodes in the motor‐related pattern, the dysconnectivity between the SMC and SPL might be a potential imaging marker that is related to motor impairment in PD patients. In the mild subtype, the global function of the motor‐related pattern, local and global function of the depression‐related pattern, and white matter microstructure were intact, which indirectly suggested that the brain impairments in the mild subtype were specifically limited.

Analyses of brain alterations in the S‐motor subtype showed impaired local function as well as global function in the motor‐related pattern, which manifested as decreased functional connectivity between the SMC and SPL within the motor‐related pattern, and disrupted functional connectivity between the motor‐related pattern and frontal–parietal regions (e.g., the inferior frontal and supramarginal gyri). The SMC and parietal cortex were disconnected, as was observed in the mild subtype. The frontal regions are always correlated with pathological alpha‐synuclein accumulation (Compta et al., 2015; Guo et al., 2018), a core pathology of PD (Braak et al., 2003) and have also been associated with motor disturbances in PD (Guo et al., 2015), which indicates that the frontal area is a target of pathological changes in PD. By combining these results with our findings, we suppose that the disrupted functional connectivity within the motor‐related pattern and frontal–parietal cortex reflect motor‐related pathology. In addition, the S‐motor subtype exhibited moderately disrupted local and global functions in the depression‐related pattern. Depression is a general neuropsychiatric symptom in PD (Nagy & Schrag, 2019). Although the S‐motor subtype was characterized by motor impairments, they might present with depression‐related symptoms, which was supported by the relatively higher depression‐related scores in the S‐motor subtype compared with normal controls.

Focusing on the S‐depression subtype, we found widespread disrupted functional connectivity both within the motor‐ and depression‐related connectivity patterns as well as outside of these two patterns, covering the frontal‐temporal and parietal‐occipital regions. Several studies demonstrated that the neural bases of depression in PD patients was likely to be diffuse, and has been referred to as disrupted structure or function in frontal, temporal and occipital regions (Feldmann et al., 2008; Gou et al., 2018; Mayberg et al., 1990; Nagy & Schrag, 2019), which corresponded with our findings and indicated serious brain damage in the S‐depression subtype. Additionally, enhanced functional connectivity in the cerebellum and thalamus was also observed in the S‐depression subtype. The cerebellum has been recognized as playing a compensatory role in PD and may help to maintain relatively normal motor function (Wu & Hallett, 2013), which may explain the lower motor scores in the S‐depression subtype. Increased functional connectivity in the thalamus observed in the S‐depression subtype corresponded with the increased metabolic activity in PD‐related spatial covariance pattern (Wu et al., 2015) and indicated increased global connectivity, which may compensate for the disrupted local function. Notably, the S‐depression subtype showed widespread disruptions in white matter microstructure manifested as increased MD in the white matter skeleton involving the superior longitudinal fasciculus, corona radiata, corpus callosum, forceps minor, and uncinate fasciculus. MD refers to the diffusion of water molecules in organic tissues and increased MD often suggests degeneration of the tissue (Sykova, 2004). Increased MD in several white matter tracts, including the superior longitudinal fasciculus, corpus callosum, and uncinate fasciculus, has been observed in PD patients (Kim et al., 2013; Surova et al., 2016), which was in agreement with our results and indicated widespread degeneration in the S‐depression subtype. Combining the widespread disrupted gray matter dysconnectivity and diffuse white matter degeneration in the S‐depression subtype, we speculate that depression aggravated brain damage and therefore may have a considerable impact on disease severity. Previous longitudinal studies reported that PD patients with depression showed a more severe cognitive decline and a faster progression of motor deterioration than nondepressed PD patients (Starkstein, Bolduc, Mayberg, Preziosi, & Robinson, 1990; Starkstein, Mayberg, Leiguarda, Preziosi, & Robinson, 1992). These studies indicated that depression aggravates the disease severity in PD, supporting our speculation.

In summary, analyses of brain alterations revealed the heterogeneity behind PD subtypes: the mild subtype showed limited dysconnectivity in the motor‐related pattern; the S‐motor subtype exhibited moderately dysfunction in the two clinically relevant patterns; the S‐depression subtype was characterized by the extensive disruption both in gray matter functional connectivity and white matter microstructure.

### 4.4 Limitations

Several limitations of the present study should be acknowledged. A fundamental issue was that this study did not include some crucial NMS of PD. Currently the included clinical domains only represented some basic but important symptoms, it would be preferable to get the patients assessed by full‐scale clinical domains (e.g., motor, depression, anxiety, sleep disorder, dysautonomia, pain, multidomain cognitive impairment, apathy, and fatigue). Hopefully, future studies will incorporate comprehensive clinical assessments and perfect the characterization of clinical heterogeneity in PD patients. Besides, whether depressive symptoms aggravate the disease severity will need to be further verified by a prospective longitudinal study.

## 5 CONCLUSION

Our study revealed heterogeneous PD subtypes according to their distinct clinically relevant connectivity features. Their functional and structural bases were shown to provide significant evidence for the unsupervised clustering procedure. Importantly, predominant depressive symptoms have a considerable impact on brain damage in PD patients.

## CONFLICT OF INTEREST

The authors have declared that no competing interest exists.

## Supporting information


**Appendix** **S1**: Supporting information

## Data Availability

The data supporting the findings of this study are available from the corresponding author upon reasonable request. They are not publicly available due to ethical restrictions.
