# Supplementary material for: Clinically relevant connectivity features define three subtypes of Parkinson's disease patients
Source: Hum Brain Mapp. 2020 Jun 26;41(14):4077–92. doi: 10.1002/hbm.25110 (PMC7469787; doi:10.1002/hbm.25110)
Supplement: Supplementary file 1 — Appendix S1: Supporting information [file HBM-41-4077-s001.docx]

Supplementary Materials

**Clinically relevant connectivity features define three subtypes of Parkinson’s disease patients**

Coauthors: Tao Guo^1#^, Xiaojun Guan^1#^, Cheng Zhou^1^, Ting Gao^2^, Jingjing Wu^1^, Zhe Song^2^, Min Xuan^1^, Quanquan Gu^1^, Peiyu Huang^1^, Jiali Pu^2^, Baorong Zhang^2^, Feng Cui^3^, Shunren Xia^4^, Xiaojun Xu^1^, Minming Zhang^1*^

^#^Tao Guo and Xiaojun Guan should be considered joint first author.

*Direct all correspondence to:

Prof. Minming Zhang, MD, PhD

Department of Radiology,

The Second Affiliated Hospital, Zhejiang University School of Medicine

No.88 Jiefang Road, Shangcheng District

Hangzhou, China, 31009

Phone: 86-0571-87315255

Fax: 86-0571-8735255

E-mail: zhangminming@zju.edu.cn

## Materials and Methods

**Rs-fMRI data analysis: preprocessing and functional connectivity analysis**

The rs-fMRI data preprocessing was performed using the Statistical Parametric Mapping version 12 (SPM, <https://www.fil.ion.ucl.ac.uk/spm/>) and Data Processing & Analysis for (Resting-State) Brain Imaging suite (<http://rfmri.org/dpabi>) (Yan, Wang, Zuo, & Zang, 2016), which included the following steps: the removal of the first 10 volumes, slice timing, realignment, spatial normalization using EPI templates, smoothing with a Gaussian kernel of 6 × 6 × 6 mm^3^ full-width at half-maximum, linear detrending, the regression of nuisance covariates (Friston 24 head motion parameters, white matter signal, and cerebrospinal fluid signal), temporal bandpass filtering (0.01-0.1 Hz), and scrubbing. Six patients and one normal control were excluded because of apparent head motion over 2 mm (translation) or 2° (rotation). Additionally, seven patients were excluded because their imaging data had more than 1/3 of the time points removed after scrubbing. Finally, 121 PD patients and 76 normal controls were included in the functional connectivity analysis.

CCA revealed two distinct connectivity patterns, including a motor-related pattern and a depression-related pattern, and each pattern comprised a set of nodes. To uncover the neurophysiological substrates of the subtypes defined by the unsupervised clustering analysis, we first calculated a functional correlation matrix to reflect the functional connectivity within each clinically relevant pattern (Figure 1C-Ⅰ), which represented the local function of this pattern. This was done by the following steps: 1) the BOLD signal time series was extracted from each node in each clinically relevant pattern by averaging across all voxels in that node; 2) the Pearson’s correlation between each pair of nodes was calculated to obtain the correlation matrix for each clinically relevant pattern. Fisher’s r-to-z transformation was applied to improve data distributions for parametric statistical analyses.

Then, to explore the global function of each clinically relevant pattern, we calculated the functional connectivity outside of each corresponding connectivity pattern (Figure 1C-Ⅱ): the connectivity pattern served as a seed, and a seed-based functional connectivity approach was used to reflect the functional connectivity between the seed (specific connectivity pattern) and the remaining voxels. Fisher’s r-to-z transformation was applied to improve data distributions for parametric statistical analyses.

**Statistical analysis**

Statistical analyses of demographic and clinical data were performed using SPSS 19.0 statistical software. The one-sample Kolmogorov-Smirnov test was used to check the normality of the data. Differences in the age, education, sex distribution, and clinical symptom scores between groups were compared with the unpaired t-tests, the Mann-Whitney U tests, and the Pearson chi-squared test as appropriate. Statistical significance was set at p < 0.05.

To reveal the predominant clinical symptoms according to the features extracted by CCA, the UPDRS III scores and HAMD scores were normalized. Paired t-tests were used to determine the predominant clinical symptom in a specific subtype.

Statistical analyses of functional connectivity representing the local and global function of a specific pattern were conducted using unpaired t-tests with age, sex, and education as covariates. Multiple comparison corrections were performed using the false discovery rate (FDR) correction with q < 0.05.

The intergroup comparisons of the white matter microstructure (FA and MD skeletons) were performed using the *Randomized* script in the FMRIB Software Library (https://fsl.fmrib.ox.ac.uk/fsl/fslwiki/), with age, sex, and education as covariates. Permutation tests with 5000 iterations and threshold-free cluster enhancement (TFCE) with a threshold of corrected p < 0.05, corrected for multiple comparisons, were performed in the intergroup comparisons.

**Supplementary Tables**

**Table S1** Comparisons of normalized clinical scores.

|  | Normalized clinical scores | | |
| --- | --- | --- | --- |
|  | Motor score  (UPDRS III) | Depression score (HAMD) | P value |
| Cluster 1 | 0.14(0.91) | 0.75 (1.13) | 0.001 |
| Cluster 2 | 0.75(0.88) | -0.37 (0.53) | <0.001 |

Data are presented as the mean (SD).

**Table S2** Comparisons of functional connectivity within the clinically relevant fiber connectivity pattern.

| ROI-ROI | p value | T value | q value |
| --- | --- | --- | --- |
| **Motor-related pattern** | | | |
| **S-depressoin < NCs** | | | |
| SMC.L-SPL.L | < 0.001 | -4.496 | 0.000718 |
| SMC.R-SPL.L | < 0.001 | -4.152 | 0.001385 |
| SMC.L-SPL.R | < 0.001 | -3.996 | 0.00167 |
| CN.L-SMC.R | < 0.001 | -3.968 | 0.001388 |
| CN.R-SMC.R | 0.001 | -3.515 | 0.005573 |
| CN.L-SMC.L | 0.001 | -3.391 | 0.00707 |
| FOC.L-SPL.R | 0.001 | -3.306 | 0.007997 |
| FMC.L-SPL.R | 0.001 | -3.270 | 0.007882 |
| SMC.R-SPL.R | 0.002 | -3.212 | 0.008444 |
| SMC.R-FMC.L | 0.002 | -3.195 | 0.008031 |
| CN.R-SMC.L | 0.002 | -3.095 | 0.010009 |
| SPL.R-SPL.L | 0.003 | -3.065 | 0.010058 |
| FMC.L-SPL.L | 0.003 | -3.057 | 0.00953 |
| FOC.L-SPL.L | 0.004 | -2.912 | 0.013745 |
| FOC.L-FMC.L | 0.005 | -2.828 | 0.016431 |
| CN.L-SPL.R | 0.006 | -2.808 | 0.016335 |
| PCN.L-SPL.R | 0.009 | -2.671 | 0.022747 |
| SMC.L-FMC.L | 0.009 | -2.663 | 0.02201 |
| PCN.R-SPL.R | 0.012 | -2.546 | 0.028777 |
| FOC.L-SMC.R | 0.014 | -2.496 | 0.031295 |
| PCN.R-SMC.L | 0.014 | -2.486 | 0.030644 |
| CN.R-SPL.R | 0.016 | -2.454 | 0.031769 |
| PCN.L-SMC.L | 0.018 | -2.404 | 0.034714 |
| PCN.L-SMC.R | 0.019 | -2.371 | 0.036238 |
| **S-motor < NCs** | | | |
| SMC.L-SPL.L | 0.001 | -3.280 | 0.032098 |
| **Mild < NCs** | | | |
| SMC.R-SPL.R | < 0.001 | -3.843 | 0.009097 |
| SMC.L-SPL.R | < 0.001 | -3.684 | 0.008017 |
| **Depression-related pattern** | | | |
| **S-depression < NCs** | | | |
| PP.R-FO.L | < 0.001 | -4.292 | 0.001615 |
| CO.R-FOC.L | < 0.001 | -3.645 | 0.008895 |
| PP.R-FOC.R | 0.001 | -3.575 | 0.007568 |
| CO.R-FMC.L | 0.001 | -3.543 | 0.006335 |
| PP.R-CO.R | 0.001 | -3.430 | 0.007427 |
| FMC.L-INS.L | 0.001 | -3.421 | 0.006383 |
| CO.R-FOC.R | 0.001 | -3.332 | 0.00735 |
| FO.L-FOC.R | 0.001 | -3.289 | 0.007403 |
| FO.L-INS.L | 0.002 | -3.090 | 0.012417 |
| CO.R-INS.L | 0.003 | -3.063 | 0.012174 |
| PP.R-FOC.L | 0.003 | -3.059 | 0.011205 |
| CO.R-FO.L | 0.003 | -3.034 | 0.011075 |
| FO.L-FMC.L | 0.003 | -3.020 | 0.010674 |
| FOC.R-FOC.L | 0.004 | -2.924 | 0.01328 |
| FOC.R-FMC.L | 0.005 | -2.890 | 0.013689 |
| FOC.L-FMC.L | 0.005 | -2.828 | 0.015404 |
| FO.L-FOC.L | 0.008 | -2.702 | 0.020871 |
| FO.L-PCN.L | 0.010 | -2.631 | 0.024026 |
| SCLC.L-PRG.L | 0.013 | -2.510 | 0.031736 |
| CO.R-PCN.L | 0.014 | -2.497 | 0.031219 |
| SCLC.L-CO.R | 0.015 | -2.475 | 0.031498 |
| SCLC.L-FO.L | 0.016 | -2.446 | 0.032505 |
| PP.R-FMC.L | 0.016 | -2.445 | 0.03114 |
| PP.R-PCN.L | 0.017 | -2.420 | 0.03186 |
| FOC.L-INS.L | 0.019 | -2.384 | 0.03362 |
| SCLC.L-INS.L | 0.023 | -2.299 | 0.040206 |
| PCN.L-INS.L | 0.028 | -2.221 | 0.047029 |
| **S-motor < NCs** | | | |
| CO.R-FOC.R | < 0.001 | -4.270 | 0.001993 |
| CO.R-FOC.L | < 0.001 | -3.815 | 0.005279 |
| CO.R-INS.L | < 0.001 | -3.797 | 0.003748 |
| PP.R-CO.R | 0.001 | -3.590 | 0.005763 |
| SCLC.L-CO.R | 0.001 | -3.587 | 0.004655 |
| PP.R-FOC.R | 0.001 | -3.393 | 0.007405 |
| PP.R-FO.L | 0.001 | -3.343 | 0.007475 |
| CO.R-FO.L | 0.002 | -3.141 | 0.012431 |
| FO.L-INS.L | 0.004 | -2.952 | 0.019652 |
| PP.R-FOC.L | 0.004 | -2.942 | 0.018206 |
| FOC.R-FOC.L | 0.010 | -2.622 | 0.041337 |

Results were corrected by false discovery rate (FDR) with q < 0.05.

Abbreviations: SPL.L, left superior parietal lobule; SPL.R, right superior parietal lobule; FMC.L, left frontal medial cortex; SMC.L, left supplementary motor cortex; SMC.R, right supplementary motor cortex; PCN.L, left precuneus; PCN.R, right precuneus; CN.L, left cuneus; CN.R, right cuneus; FOC.L, left frontal orbital cortex; FOC.R, right frontal orbital cortex; INS.L, left insula; PRG.L, left precentral gyrus; FO.L, left frontal operculum; CO.R, right central opercular; PP.R, right planum polare; SCLC.L, left supracalcarine cortex.

**Table S3** Comparisons of functional connectivity between motor-related pattern and the remaining voxels in the brain

| Cluster Index | Region | Hemisphere | Cluster size | Peak MNI coordinate | | | Peak T value |
| --- | --- | --- | --- | --- | --- | --- | --- |
|  |  |  |  | x | y | z |  |
| **S-depression < NCs** | | | | | | | |
| 1 | Frontal_Inf_Tri | L | 32 | -42 | 21 | 0 | -3.7769 |
| 2 | Frontal_Inf_Oper | L | 17 | -51 | 3 | 6 | -3.93 |
| 3 | Frontal_Mid | R | 10 | 18 | 48 | -24 | -3.5257 |
| 4 | Precentral | R | 15 | 48 | -9 | 60 | -3.949 |
| 5 | Supp_Motor_Area | L | 18 | -6 | 3 | 57 | -3.7431 |
| 6 | Postcentral | R | 19 | 63 | -12 | 45 | -4.2715 |
| 7 | Frontal_Sup | R | 12 | 15 | -3 | 75 | -3.9115 |
| 8 | Temporal_Pole_Sup | R | 91 | 63 | 6 | 0 | -4.2342 |
| 9 | Temporal_Sup | R | 54 | 66 | -33 | 12 | -3.9721 |
| 10 | Temporal_Mid | L | 79 | -57 | 3 | -15 | -4.4795 |
| 11 | Temporal_Pole_Mid | R | 32 | 51 | 18 | -27 | -4.0619 |
| 12 | Fusiform | L | 18 | -27 | -6 | -45 | -4.0582 |
| 13 | Parietal_Sup_L | L | 422 | -42 | -39 | 30 | -6.1899 |
| 14 | Parietal_Sup | R | 49 | 15 | -57 | 72 | -4.4426 |
| 15 | Parietal_Inf | R | 27 | 27 | -45 | 54 | -3.8338 |
| 16 | SupraMarginal | R | 18 | 57 | -30 | 39 | -3.5192 |
| 17 | Occipital_Inf | R | 151 | 42 | -90 | -9 | -4.3485 |
| 18 | Occipital_Mid | L | 458 | -27 | -81 | 15 | -4.9118 |
| 19 | Cuneus | R | 13 | 9 | -99 | 18 | -3.954 |
| 20 | Occipital_Mid | R | 11 | 33 | -69 | 3 | -3.697 |
| 21 | Cingulum_Mid | L | 10 | -9 | 9 | 36 | -3.8361 |
| 22 | Cingulum_Mid | L | 22 | -12 | -21 | 42 | -3.9189 |
| 23 | Cingulum_Mid | R | 17 | 9 | 12 | 39 | -4.5091 |
| 24 | Cerebelum_Crus1 | R | 25 | 48 | -42 | -33 | -4.5786 |
| 25 | Cerebelum_Crus1 | L | 20 | -57 | -48 | -30 | -3.9715 |
| **S-depression > NCs** | | | | | | | |
| 26 | Cerebellum_9 | R | 103 | 9 | -57 | -48 | 4.3163 |
| 27 | Cerebellum_Crus2 | L | 23 | -39 | -84 | -42 | 3.8496 |
| 28 | Cerebelum_Crus1 | R | 12 | 27 | -75 | -30 | 3.6803 |
| 29 | Thalamus | L | 176 | -6 | -6 | 9 | 4.2727 |
| **S-motor < NCs** | | | | | | | |
| 1 | Frontal_Inf_Oper | L | 18 | -42 | 9 | 12 | -4.9703 |
| 2 | Insular | L | 10 | -33 | -12 | 24 | -4.9801 |
| 3 | SupraMarginal | L | 11 | -45 | -42 | 27 | -4.8924 |

**Table S4** Comparisons of functional connectivity between depression-related pattern and the remaining voxels in the brain

| Cluster Number | Region | Hemisphere | Cluster size | Peak MNI coordinate | | | Peak T value |
| --- | --- | --- | --- | --- | --- | --- | --- |
|  |  |  |  | x | y | z |  |
| **S-depression < NCs** | | | | | | | |
| 1 | Frontal_Mid | R | 79 | 30 | 48 | 15 | -3.8919 |
| 2 | Frontal_Inf_Tri | L | 69 | -39 | 27 | 9 | -3.9816 |
| 3 | Frontal_Sup | R | 41 | 15 | -6 | 78 | -4.6503 |
| 4 | Frontal_Inf_Tri | R | 40 | 39 | 30 | 0 | -3.9466 |
| 5 | Frontal_Mid | R | 26 | 18 | 48 | -24 | -4.1364 |
| 6 | Precentral | R | 10 | 48 | 0 | 39 | -3.6887 |
| 7 | Temporal_Sup | R | 145 | 66 | -33 | 9 | -4.4249 |
| 8 | Rolandic_Oper | R | 106 | 66 | 3 | 6 | -3.8832 |
| 9 | Rolandic_Oper | L | 12 | -48 | 3 | 12 | -3.6511 |
| 10 | Temporal_Mid | L | 77 | -57 | 0 | -15 | -4.7927 |
| 11 | Temporal_Pole_Mid | R | 27 | 51 | 18 | -27 | -4.3764 |
| 12 | Fusiform | L | 12 | -30 | -3 | -45 | -3.8901 |
| 13 | Fusiform | R | 15 | 30 | -84 | -6 | -3.4881 |
| 14 | Parietal_Inf | L | 19 | -27 | -42 | 39 | -4.2147 |
| 15 | Precuneus | L | 15 | -15 | -48 | 57 | -3.5799 |
| 16 | Parietal_Sup | L | 73 | -27 | -54 | 69 | -4.1576 |
| 17 | Parietal_Sup | R | 152 | 18 | -60 | 69 | -4.7319 |
| 18 | SupraMarginal | L | 229 | -42 | -39 | 30 | -5.4161 |
| 19 | Occipital_Mid | L | 487 | -24 | -81 | 15 | -4.712 |
| 20 | Occipital_Sup | R | 79 | 24 | -84 | 12 | -4.4239 |
| 21 | Occipital_Inf | R | 22 | 42 | -90 | -9 | -3.7714 |
| 22 | Occipital_Sup | L | 29 | -9 | -90 | 39 | -4.5458 |
| 23 | Cuneus | R | 12 | 9 | -99 | 18 | -3.7453 |
| 24 | Lingual | R | 18 | 9 | -54 | 0 | -4.0262 |
| 25 | Insula | L | 52 | -39 | 15 | -3 | -4.4106 |
| 26 | Cingulum_Ant | L | 10 | -9 | 30 | 15 | -3.508 |
| 27 | Cingulum_Ant | R | 21 | 9 | 30 | 18 | -4.2471 |
| 28 | Cingulum_Mid | R | 157 | 9 | 12 | 39 | -4.902 |
| 29 | Cerebellum_Crus1 | R | 22 | 45 | -39 | -33 | -4.2755 |
| **S-depression > NCs** | | | | | | | |
| 30 | Cerebellum_9 | R | 102 | 9 | -57 | -48 | 3.996 |
| 31 | Cerebellum_Crus2 | R | 75 | 36 | -78 | -48 | 3.9776 |
| 32 | Cerebellum_Crus1 | R | 50 | 27 | -72 | -30 | 3.9627 |
| 33 | Vermis_9 | - | 12 | -3 | -57 | -30 | 3.4322 |
| 34 | Thalamus | L | 36 | -6 | -6 | 9 | 3.8502 |
| 35 | Precuneus | R | 28 | 9 | -63 | 42 | 3.9078 |
| **S-motor < NCs** | | | | | | | |
| 1 | Frontal_Inf_Oper | L | 412 | -42 | 6 | 12 | -6.2723 |
| 2 | Temporal_Mid | L | 13 | -48 | -15 | -9 | -4.1235 |
| 3 | Temporal_Sup | R | 56 | 51 | -15 | -6 | -4.7886 |
| 4 | Rolandic_Oper | R | 37 | 60 | 3 | 15 | -4.3537 |
| 5 | Parietal_Inf | L | 13 | -27 | -42 | 39 | -4.1538 |
| 6 | SupraMarginal | R | 172 | 60 | -30 | 33 | -4.9337 |
| 7 | Occipital_Mid | L | 80 | -30 | -96 | -3 | -4.2147 |
| 8 | Occipital_Inf | R | 72 | 39 | -87 | -6 | -4.0328 |
| 9 | Occipital_Mid | L | 11 | -21 | -90 | 15 | -3.6285 |
| 10 | Cingulum_Mid | R | 216 | 3 | 9 | 42 | -4.8822 |
| 11 | Cingulum_Ant | L | 23 | -3 | 30 | -3 | -4.2763 |
| 12 | Insular | R | 123 | 30 | 24 | 3 | -4.6925 |

**Table S5** Differences in mean diffusivity (MD) between S-depression patients and normal controls.

| Cluster Index | Voxels | Peak p | Peak MNI coordinate | | | Regions |
| --- | --- | --- | --- | --- | --- | --- |
|  |  |  | x | y | z |  |
| 1 | 5728 | 0.026 | 19 | 10 | 41 | SLF_Bi, SCR_R, ACR_Bi, CC_genu/body, FM, UF_Bi, IFOF_Bi, ATR_Bi, Cingulum_Bi |
| 2 | 1517 | 0.04 | -26 | -20 | 53 | SLF_L, CPT_L |
| 3 | 1049 | 0.047 | -36 | -37 | 25 | SLF_L |
| 4 | 214 | 0.047 | 33 | 4 | 42 | SLF_R |
| 5 | 113 | 0.049 | -54 | -31 | 36 | SLF_L |
| 6 | 31 | 0.049 | 26 | 6 | 35 | SLF_R |

Abbreviations: SLF, superior longitudinal fasciculus; SCR, superior corona radiata; ACR, anterior corona radiata; CC, corpus callosum; FM, forceps minor; UF, uncinate fasciculus; IFOF, inferior fronto-occipital fasciculus; ATR, anterior thalamic radiation; R, right; L, left; Bi, bilateral.

**Table S6** Clinical scale scores for dataset-1 and dataset-2

|  | dataset-1 | dataset-2 | P value |
| --- | --- | --- | --- |
| UPDRS III,  mean ± SD (min, max, median) | 25.76 ± 13.33  (5, 67, 23) | 19.04 ± 10.83  (4, 49, 16) | < 0.001 |
| MMSE,  mean ± SD (min, max, median) | 26.77 ± 3.70  (12, 30, 28) | 26.86 ± 3.95  (11, 30, 28) | 0.337 |
| HAMD,  mean ± SD (min, max, median) | 6.16 ± 5.36  (0, 31, 5) | 6.60 ± 5.80  (0, 27, 5) | 0.844 |
| HAMA,  mean ± SD (min, max, median) | 5.53 ± 4.90  (0, 25, 4) | 6.60 ± 5.05  (0, 25, 6) | 0.049 |
| ESS,  mean ± SD (min, max, median) | 5.64 ± 5.18  (0, 24, 4) | 6.27 ± 5.12  (0, 20, 5) | 0.251 |
| PDQ-39,  mean ± SD (min, max, median) | 26.72 ± 20.03  (0, 85, 20.5) | 23.46 ± 20.63  (0, 96, 16.5) | 0.139 |

**Table S7** Demographic information and clinical scale scores of dataset-2

|  | Parkinson’s disease patients | Subtypes of Parkinson’s disease patients | | | Comparisons | | | |
| --- | --- | --- | --- | --- | --- | --- | --- | --- |
|  |  | Cluster 1  (Mild, n=63) | Cluster 2  (S-motor, n=24) | Cluster 3  (S-depression, n=11) | Comparisons among PD groups  (p value) | post-hoc (p value) | | |
|  |  |  |  |  |  | Cluster 1 vs. Cluster 2 | Cluster 1 vs. Cluster 3 | Cluster 2 vs. Cluster 3 |
| **Sex**, M/F | 60/38 | 38/25 | 18/6 | 4/7 | 0.091 | - | - | - |
| **Age**, mean ± SD (min, max, median) | 59.99 ± 10.13  (32.71, 82.05, 61.62) | 59.04 ± 10.69  (32.71, 78.59, 60.88) | 62.50 ± 8.86  (47.54, 79.60, 63.65) | 59.96 ± 9.21  (50.81, 82.05, 56.97) | 0.366 | - | - | - |
| **Education**, mean ± SD (min, max, median) | 8.26 ± 4.75  (0, 18, 8) | 8.02 ± 5.14  (0,18, 8) | 9.65 ± 4.00  (4, 18, 9) | 6.55 ± 3.21  (1, 11, 8) | 0.164 | - | - | - |
| **Duration**, mean ± SD (min, max, median) | 4.77 ± 4.08  (0.22, 30.96, 4.01) | 4.18 ± 2.90  (0.22, 14.23, 3.61) | 6.66 ± 6.34  (1.15, 30.96, 5.22) | 3.99 ± 2.43  (0.99, 9.08, 3.65) | 0.031 | 0.032 | - | - |
| **GCO**, mean ± SD (min, max, median) | 0 ± 2.71  (-4.82, 9.00, -0.32) | -1.49 ± 1.59  (-4.82, 1.92, -1.84) | 2.63 ± 2.43  (-1.73, 9.00, 2.33) | 2.79 ± 1.63  (0.35, 5.01, 2.48) | < 0.001 | < 0.001 | < 0.001 | - |
| **PDQ39**, mean ± SD (min, max, median) | 23.46 ± 20.63  (0, 96, 16.5) | 16.98 ± 15.79  (0, 96, 13) | 33.21 ± 22.06  (0, 84, 35.5) | 39.27 ± 26.38  (9, 88, 33) | < 0.001 | 0.002 | 0.001 | - |
| **UPDRS III,** mean ± SD (min, max, median) | 19.04 ± 10.83  (4, 49, 16) | 16.51 ± 9.24  (4, 41, 13) | 28.46 ± 11.10  (9, 49, 26) | 13.00 ± 5.68  (6, 25, 14) | < 0.001 | < 0.001 | - | < 0.001 |
| **HY**,  median, range | 2, 1~3 | 2, 1~3 | 2.5, 1.5~3 | 2, 1~2.5 | 0.011 | 0.003 | 0.746 | 0.078 |
| **MMSE**, mean ± SD (min, max, median) | 26.86 ± 3.95  (11, 30, 28) | 26.27 ± 4.57  (11, 30, 28) | 28.21 ± 1.38  (26, 30, 28) | 27.27 ± 3.29  (19, 30, 28) | 0.563 | - | - | - |
| **HAMD**, mean ± SD (min, max, median) | 6.60 ± 5.80  (0, 27, 5) | 4.08 ± 3.74  (0, 18, 3) | 9.17 ± 5.66  (0, 22, 7.5) | 15.45 ± 4.93  (7, 27, 15) | < 0.001 | < 0.001 | < 0.001 | 0.003 |
| **HAMA**, mean ± SD (min, max, median) | 6.60 ± 5.05  (0, 25, 6) | 4.76 ± 3.67  (0, 15, 4) | 8.00 ± 4.68  (0, 19, 7) | 14.09 ± 5.09  (8, 25, 13) | < 0.001 | 0.004 | < 0.001 | < 0.001 |
| **ESS**, mean ± SD (min, max, median) | 6.27 ± 5.12  (0, 20, 5) | 4.68 ± 3.97  (0, 14, 3) | 9.88 ± 5.95  (3, 20, 8.5) | 7.45 ± 5.13  (1, 15, 8) | < 0.001 | < 0.001 | - | - |

**Supplementary Figure legends**


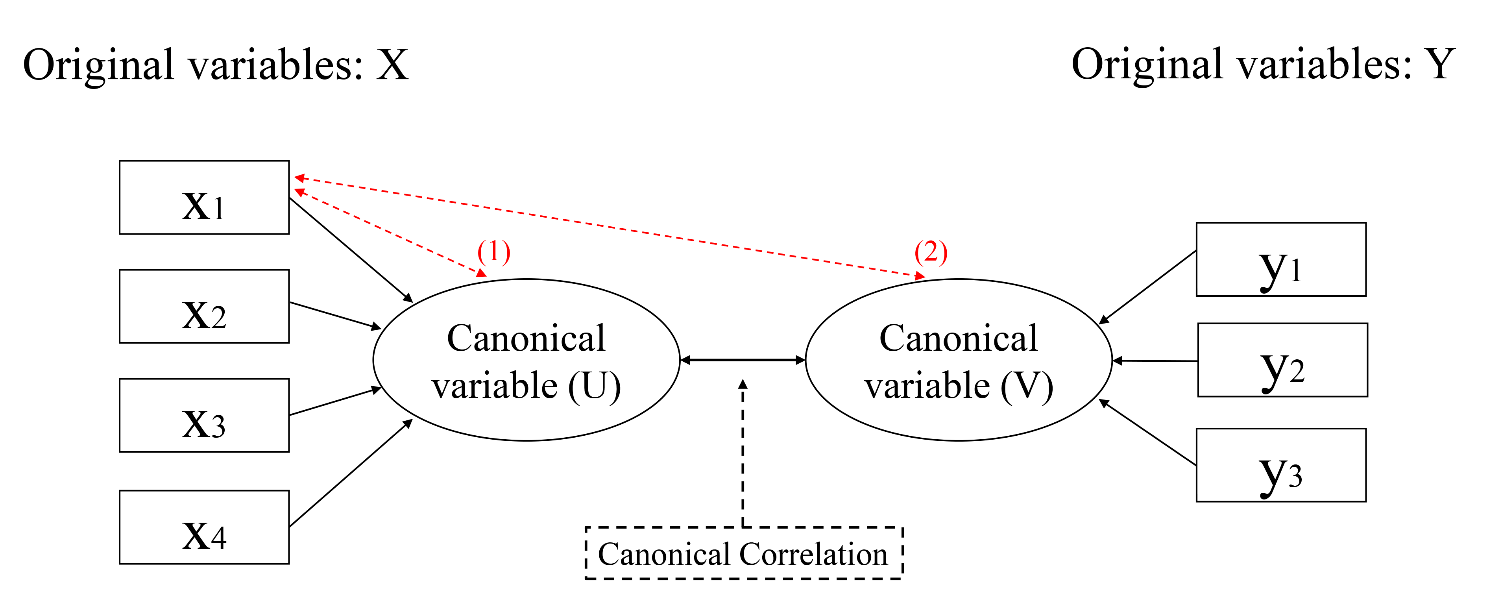


**Figure S1** Illustration of the canonical correlation analysis (CCA). CCA aims to investigate underlying relationships between two sets of variables (X and Y). Each significant CCA mode identifies a linear combination (canonical variable, U) of a set of original variables (X), and a linear combination (canonical variable, V) of another set of original variables (Y), where the correlation between these two canonical variable (U and V) is maximal. Canonical loading (1) is the correlation between an original variable and its corresponding canonical variable. Cross loading (2) depicts the correlation between an original variable and the canonical variable of another set. Squared loading (canonical or cross loading) indicates the amount of variance of a variable explained by the canonical variate.


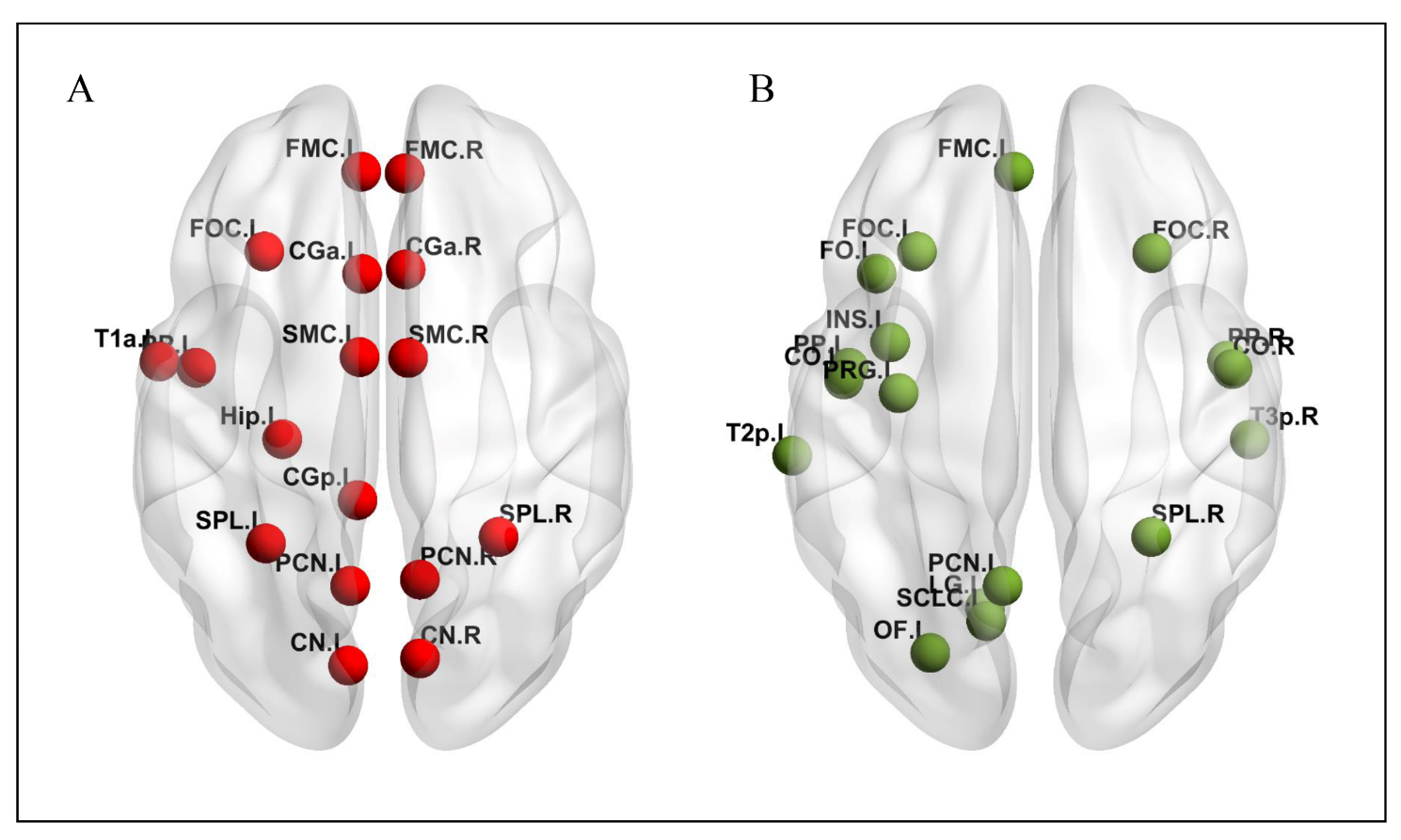


**Figure S2** Squared connectivity score loadings are summarized by depicting the neuroanatomical distribution of the top 20% ROIs with the largest R^2^ values, summed across all connectivity features associated with a given node. (A) motor-related connectivity pattern, (B) depression-related connectivity pattern. Abbreviations: CN.L, left cuneus; CN.R, right cuneus; CO.L, left central operculum; CO.R, right central operculum; CGa.L, left cingulate gyrus, anterior division; CGa.R, right cingulate gyrus, anterior division; CGp.L, left cingulate gyrus, posterior division; FMC.L, left frontal medial cortex; FMC.R, right frontal medial cortex; FOC.L, left frontal orbital cortex; FOC.R, right frontal orbital cortex; FO.L, left frontal operculum; Hip.L, left hippocampus; INS.L, left insula; LG.L, left lingual gyrus; OF.L, left occipital fusiform gyrus; PCN.L, left precuneus; PCN.R, right precuneus; PP.L, left planum polare; PP.R, right planum polare; PRG.L, left precentral gyrus; SPL.L, left superior parietal lobule; SPL.R, right superior parietal lobule; SMC.L, left supplementary motor cortex; SMC.R, right supplementary motor cortex; SCLC.L, left supracalcarine cortex; T1a.L, left superior temporal gyrus, anterior division; T2p.L, left middle temporal gyrus, posterior division; T3p.R, inferior temporal gyrus, posterior division.


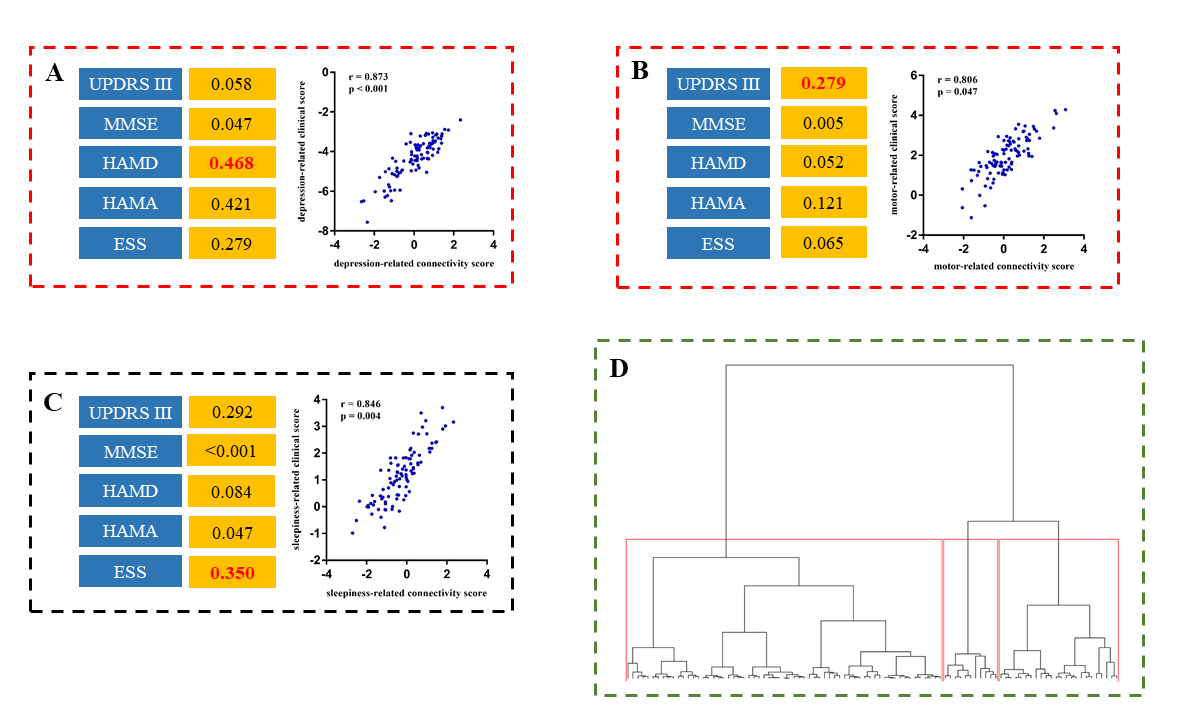


**Figure S3** CCA and hierarchical clustering define three connectivity-based subtypes in dataset-2. CCA was used to define a low-dimensional representation of clinically relevant connectivity features and identified a “depression-related” pattern (A), “motor-related” pattern (B) and a “sleepiness-related pattern” (C). The scatterplots in A, B and C illustrated the correlation between connectivity component and clinical component for depression-related pattern (r = 0.873, p < 0.001), motor-related pattern (r = 0.806, p = 0.047), and sleepiness-related pattern (r = 0.846, p = 0.004), respectively. To the left of each scatterplot, squared cross loadings for clinical scores were depicted. (D) Hierarchical clustering analysis. According to the depression-related and motor-related connectivity scores, patients with Parkinson’s disease were assigned into three subtypes.
